# Diode area melting of Ti6Al4V: effects of multi-laser processing on microstructure and residual stress

**DOI:** 10.1007/s40964-026-01951-x

**Published:** 2026-09-08

**Authors:** Anqi Liang, Sarath Veetil, Kristian Groom, Kamran Mumtaz

**Affiliations:** School of Mechanical, Aerospace, and Civil Engineering, University of Sheffield, S1 3JD Sheffield, UK

**Keywords:** Diode area melting, Additive manufacturing, Residual stress, Ti6Al4V, Multiple diode lasers

## Abstract

Laser Powder Bed Fusion (LPBF) is a widely adopted additive manufacturing technique for producing geometrically complex metallic components. While this process yields fine microstructures that are beneficial for strength, the associated rapid thermal cycles generate steep temperature gradients and high residual stresses, which may lead to warping, cracking, and dimensional instability. Diode Area Melting (DAM) employs arrays of low-power diode lasers operating at a wavelength of 808 nm. The diode beams are integrated into a customised optical head that traverses the powder bed in the *X*–*Y* plane, enabling a broader thermal interaction area and reducing local cooling rate. In this study, single-layer Ti6Al4V samples were processed by DAM using 3, 4, and 5 aligned 808 nm diode lasers, each operating at 5 W. The effects of laser number and scanning speed (100, 150, 200, and 250 mm/min) on surface roughness, melt pool geometry, microstructure, simulated cooling rate, and residual stress were systematically investigated. DAM produced lower simulated cooling rates (2211–8400 K/s) than those typically reported for LPBF (10⁴–10⁷ K/s), leading to coarser HCP lath spacings (0.8–1.3 μm) and evidence of retained *β*. Near-surface tensile residual stresses of up to 138.2 MPa were measured. The simulated lower cooling rates and reduced residual stress indicate that DAM may provide improved control over solidification behaviour and stress evolution.

## Introduction

Laser powder bed fusion (LPBF) has attracted considerable interest in both industry and academia because of its advantages, including design flexibility, the ability to fabricate metal components with complex geometries, and its unique thermal history and features [1]. LPBF typically employs a galvo mirror system to rapidly reflect high-power fibre lasers or solid-state lasers (e.g. Nd:YAG), across successive thin layers of metal powder [2]. Diode area melting (DAM) is a novel alternative to LPBF that processes metals using an array of low-power diode laser emitters [3]. The main differences between DAM and LPBF are as follows: First, DAM replaces the galvo system, which reflects a single fibre laser, with a gantry system that moves in *x-*/*y-* directions to maintain multiple laser beams in parallel. Second, DAM utilises diode lasers as a more efficient energy source due to their shorter wavelength compared to fibre lasers, which is particularly advantageous for highly reflective materials. For example, the absorptivity of Ti6Al4V under 450 nm diode laser was reported to be approximately 14% higher than that under 1064 nm fibre laser [4]. Third, DAM employs multiple diode lasers arranged in a customised array instead of a single fibre laser, enabling larger areas to be irradiated simultaneously. The layer-by-layer 2D geometry can be tailored by selectively activating lasers at specific locations [3], providing additional spatial control over the melting pattern.

Recent studies have demonstrated the feasibility of DAM for processing a range of metallic materials. Early work successfully melted a full layer of Sn powder using a hybrid energy source comprising a diode laser array and a Q-switched pulsed laser [5]. Zavala-Arredondo et al. [6] processed 316L stainless steel (SS) with 19 aligned 808 nm diode lasers (total beam area = 1.9 mm × 0.1 mm in a linear alignment of 1 × 19) and achieved a relative density above 99% at an energy density of 86 J/mm^3^. Their thermal simulations indicated that DAM could substantially reduce the temperature gradient (*G*) and cooling rate. Another study reported that the sub-grain cellular size of DAM-processed 316L stainless steel was approximately 100% larger than that of LPBF-fabricated material, which was attributed to the slower cooling rate [7]. The fabrication of 17-4PH stainless steel was also investigated using DAM with 19 diode lasers (total laser power of 50 W) [3]. However, with a layer thickness of 150 µm, the relative density was only 79.18% and lack-of-fusion porosity was observed due to inconsistencies in the manual powder-deposition process.

Several studies have investigated the DAM processing of Ti6Al4V alloys. Aydin et al. [8] reported that the low-power 450 nm diode lasers produced slower solidification and promoted the formation of a stable *α* + *β* microstructure with larger grain size. Caglar et al. [9] achieved near-full density Ti6Al4V components with a relative density above 99% using multiple 450 nm diode lasers and observed a coarser lamellar morphology than that typically reported for LPBF materials. Alsaddah et al. [4] compared 450 nm and 808 nm diode lasers for processing Ti6Al4V and they found that the absorption at 450 nm lasers was 11% higher than that with 808 nm lasers. Their work also reported that DAM cooling rates (1000–1670 °C/s) were significantly lower than those commonly reported for LPBF (~ 10^7^ K/s), thereby favouring the retention of the *β* phases.

Residual stress remains one of the main challenges in LPBF-fabricated components due to rapid melting and solidification during repeated heating and cooling cycles [10]. Ahmed et al. [11] measured residual stresses in LPBF Ti6Al4V and found that tensile stress could exceed 900 MPa at the component edge, nearly reaching the yield strength. Machirori et al. [12] modelled the spatial and temporal evolution of residual stress in LPBF Ti6Al4V and reported tensile residual stresses of 800–920 MPa in the scanning direction and 500–760 MPa in the transverse direction. Such stresses cause warpage and cracking and may promote failure when combined with service stresses, particularly under tensile loading [13]. Mercelis et al. [14] described residual stress formation in LPBF using the temperature gradient mechanism (TGM) and the cool-down mechanism. During heating, irradiated material expands but is constrained by the surrounding material, leading to compressive stress. During cooling, the shrinkage is again constrained, resulting in residual tensile stress. Although residual stress development in LPBF has been extensively investigated [15–17], corresponding studies on DAM-processed materials remain limited. Given the substantially lower cooling rates associated with DAM, residual stress development may differ significantly from that observed in LPBF.

Ti6Al4V is one of the most widely used titanium alloys because of its high specific strength, corrosion resistance, and biocompatibility. It is widely applied in aerospace [18], automotive [19], and biomedical applications [20]. Previous studies on DAM processing of Ti6Al4V have primarily focused on densification and process-parameter optimisation, whereas quantitative analysis of near-surface residual stress and its correlation with local solidification behaviour remains limited. The present study investigated the DAM-built Ti6Al4V specimens by employing multiple 808 nm diode lasers. Although previous studies have reported higher absorptivity for 450 nm diode lasers than for 808 nm diode lasers, the use of 808 nm diode lasers as the laser source in a multi-laser DAM system remains scientifically worthy of investigation. The objective was to investigate residual stress development and examine how the measured stress trend relates to simulated cooling behaviour, microstructural characteristics, and phase constitution with different laser configurations and process conditions, rather than to compare the different laser wavelengths. Therefore, single-layer Ti6Al4V specimens were processed using three, four, and five 808 nm diode lasers at different scanning speeds. The different laser configurations introduced complex multi-beam interactions, including thermal-field superposition, overlap between neighbouring irradiated regions, together with the resulting cumulative heating. The influence of these interactions on thermal history, microstructural evolution, and residual stress has not yet been systematically investigated for 808 nm DAM processing. The single-layer configuration was selected to isolate intra-layer thermal, microstructural, and residual-stress responses. Although the results cannot be directly extrapolated to 3D multilayer components, the trends can still provide a baseline for future investigation of repeated thermal cycling and stress accumulation in multilayer DAM builds.

## Methodology

### Experiment setup

**Fig. 1:**
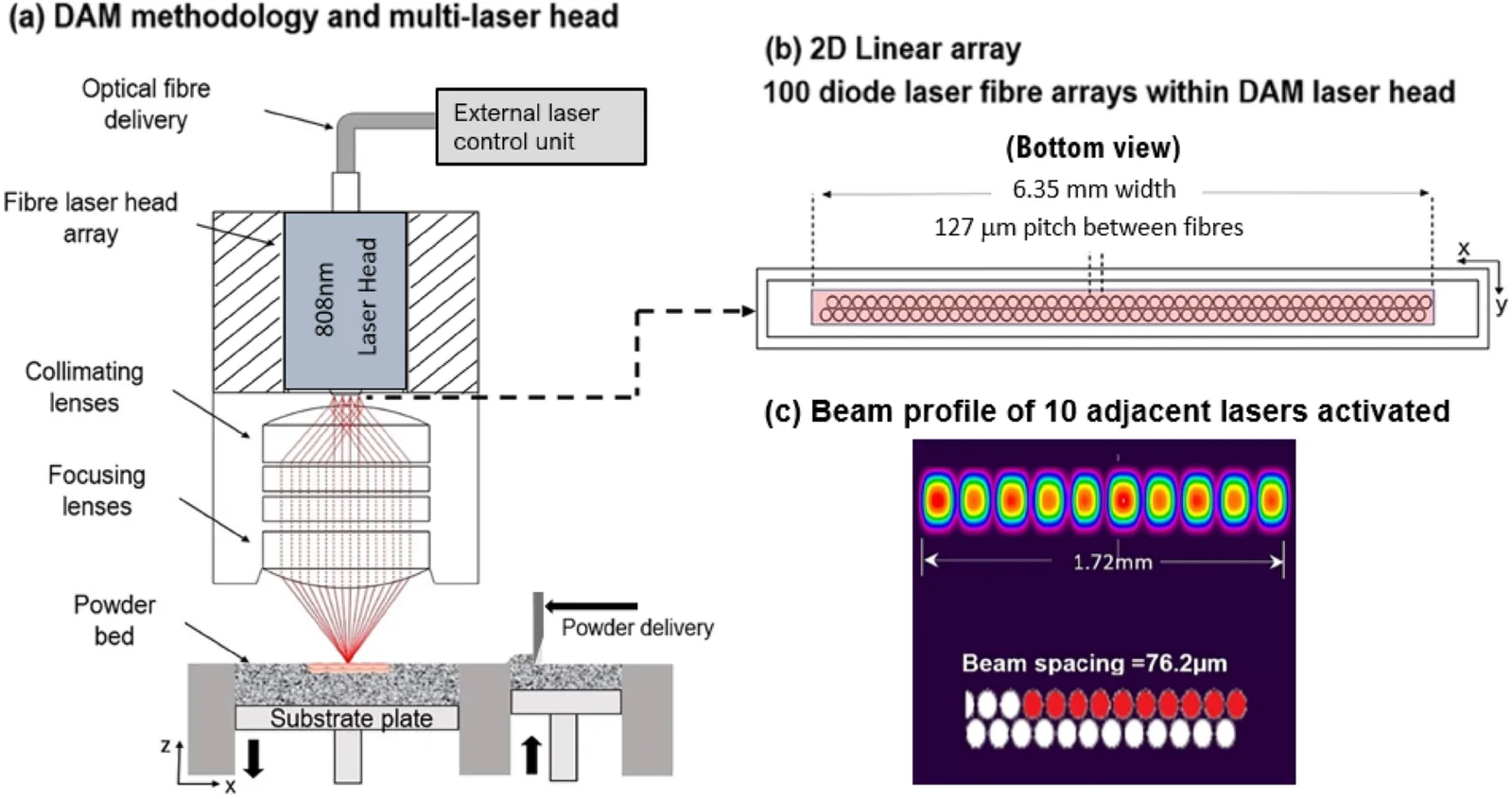
**a** Schematic of testing rig, **b** bottom view of the multiple laser beam configuration, and **c** beam profile of 10 adjacent lasers activated [21]

DAM processing was conducted using a novel system developed at the University of Sheffield [21], as shown in Fig. 1. Figure 1a presents a schematic of the experimental rig. The powder bed, powder feedstock and powder delivery system are identical to those in conventional LPBF systems. The novelty of DAM lies in the optical system, where 100 optical fibres are coupled into a custom-made laser head, enabling the integration of up to 100 diode lasers as the laser source. The distribution of 100 fibres is shown as Fig. 1b, they are arranged in two compact linear arrays of 50 fibres with a centre-to-centre spacing of 127 µm between. Adjacent fibres. A set of optical lenses is used to collimate and focus the laser beams at a focal length of 6.35 mm. In this study, low-power diode lasers with a wavelength of 808 nm and a maximum output power of 5 W per laser were used in this study. At the focal plane, each laser beam exhibited an individual Gaussian intensity profile with a spot size of 65 µm. Owing to the × 0.6 magnification factor produced by the lens system, the centre-to-centre spacing between adjacent beams was reduced to 76.2 µm, as shown in Fig. 1c.

### Specimen manufacturing procedure

Gas-atomised Ti6Al4V powder with a particle size range of 15–45 µm was supplied by Carpenter Additive under the trade name *PowderRange Ti64*. The chemical composition specified by the manufacturer is listed in Table 1.

**Table 1:** Chemical composition (wt%) of Ti6Al4V alloy powders [22]

Ti	Al	V	Fe	O	C	N	H	Y
Bal	5.5–6.5	3.5–4.5	0.25	0.13	0.08	0.03	0.0125	0.005

Prior to processing, the DAM chamber was purged with argon to reduce the oxygen concentration and minimise oxidation. Once the oxygen concentration fell below 0.1%, the purge rate was reduced to maintain a stable low-oxygen environment during scanning. The multi-laser head was controlled using Lightburn software to traverse above the powder bed in the *x*-/*y*- directions. The powder bed layer thickness was set to 1 mm to provide sufficient material for laser interaction while preventing bonding between the specimen and the substrate, which could affect the residual stress distribution. Under the investigate conditions, the melt pool did not reach the substrate, allowing the single-layer specimens to be removed easily after processing.

The processing parameters are summarized in Table 2. Four scanning speeds, corresponding to the traverse speed of the multi-laser head, were investigated: 100, 150, 200, and 250 mm/min. Three, four, and five lasers were activated, corresponding to the total laser powers of 15, 20, and 25 W, respectively. A constant hatch spacing (*h*) of 200 µm, defined as the distance between adjacent scan tracks, was applied for all specimens. To provide a nominal comparison of the energy supplied per unit processed area, the nominal areal energy density was calculated as *E*_*area*_ = *P*/*(v × h),* where *P* is the total laser power, *v* is the scanning speed and *h* is the hatch spacing. As the present study was limited to single-layer specimens and this metric was intended to compare the nominal energy supplied per unit scanned area, the powder layer thickness was not included in the calculation. The calculated values, listed in Table 2, were used only as nominal comparative parameters rather than locally absorbed energy densities.

**Table 2:** Parameters for processing Ti6Al4V alloy

No. of lasers	Total laser power, *P* (W)	Scanning speed, *v* (mm/min)	Hatch spacing, *h* (µm)	Nominal areal energy density, *E*_*area*_ (J/mm^2^)
3 lasers	15	100	200	45
		150		30
		200		22.5
		250		18
4 lasers	20	100	200	60
		150		40
		200		30
		250		24
5 lasers	25	100	200	75
		150		50
		200		37.5
		250		30

**Fig. 2:**
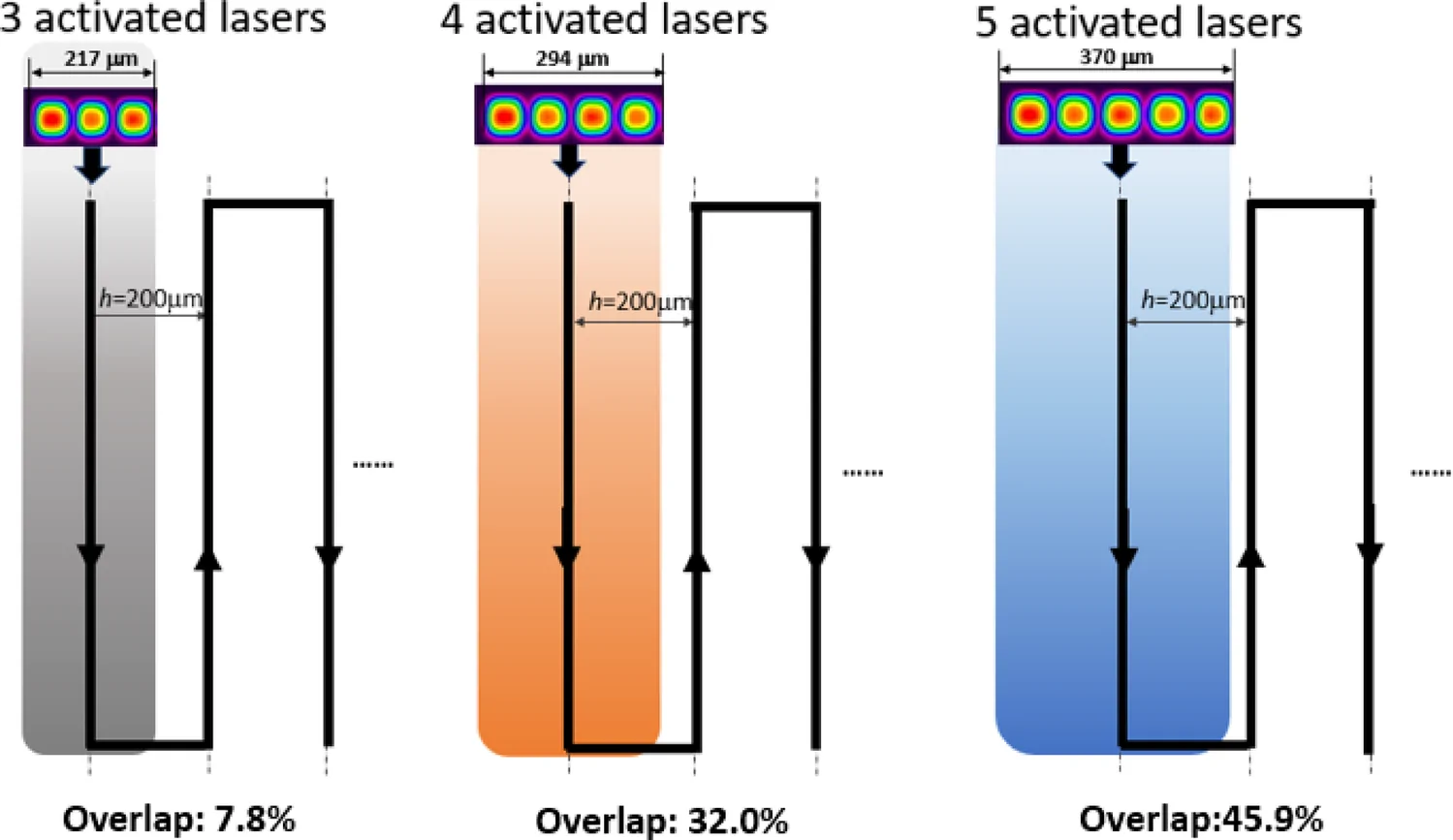
Schematic of scanning strategies with **a** 3 lasers, **b** 4 lasers, and **c** 5 lasers. The arrows represent the scanning direction of each path

The scanning strategy is illustrated schematically in Fig. 2. The combined beam width varied with the number of activated lasers: 217 µm for three lasers, 294 µm for four lasers and 370 µm for five lasers. The track overlap ratio was calculated as: (beam width—hatch spacing) ÷ beam width, and the calculated values are shown below the corresponding schematics in Fig. 2. For each processing condition, three 10 mm × 10 mm single-layer samples were fabricated.

### Microstructure analysis

Surface topography was measured using an Alicona InfiniteFocusSL 3D optical profilometer based on focus-variation technology, with the colour scale representing relative surface height. An area of approximately 2 × 2 mm^2^ at the centre of each specimen was measured. Five square-spiral profile paths were randomly selected at difference positions within the measured area. Each path had a total evaluation length of approximately 20 mm and a profile width of 10 µm. A surface roughness value (*Ra*) was calculated for each path. Roughness filtering was applied using a cut-off length of 8 mm. The use of square-spiral profiles reduced the influence of the laser scan-track direction on the roughness measurement. The reported *Ra* value represents the average of the five measurements, and the corresponding standard deviation is shown by the error bars.

To prepare cross-sections for microstructural characterization, the specimens were cut using a Buehler Isomet 5000 precision saw equipped with a rotating blade operating at 2300 rpm and a feed rate of 0.15 mm/s. The sections were then mounted in PolyFast resin, a conductive thermosetting phenolic compound, using a Buehler Simplimet 1000 mounting press. Mounting was performed at 180 °C for 4 min, followed by 2 min of cooling. To achieve a mirror-like surface finish, the mounted specimens were sequentially ground with *#*240,* #*600, *#*1200, *#*2500 and *#*4000 abrasive papers and then polished using 1 μm diamond paste.

Melt pools were observed using a Nikon LV150NL optical microscope (OM), and the microstructure was characterised using a TESCAN VEGA3 scanning electron microscope (SEM). Melt pool dimensions and heat affected zone dimensions were measured using Image J software, and five measurements were taken at random selected locations in each image to calculate the average. Electron backscattered diffraction (EBSD) was used to investigate grain morphology, phase distribution, and crystallographic orientation using an Oxford Instruments® C-Nano EBSD detector operated at 20 kV. The step size was adjusted according to the scanning area to balance spatial resolution and acquisition time, with 0.1 μm at 650 × magnification. For EBSD analysis, final polishing was performed using a suspension of 90 vol% colloidal silica and 10 vol% hydrogen peroxide. EBSD data were analysed with AztecCrystal software (Oxford Instruments), followed by a CI threshold of 0.1 and a nearest-neighbour cleanup procedure. Isolated incorrectly indexed pixels and unindexed pixels were replaced and filled with at least four indexed pixels surrounding them by using level 5 of Zero Solution Removal function. A misorientation angle of 15° was used to define grain boundaries. Grain morphology and size were assessed by calculating grain aspect ratios and average grain size.

X-ray diffraction (XRD) was used for phase identification using a PANalytical X’Pert^3^ Powder diffractometer (Malvern Panalytical) with Cu Kα radiation (*λ* = 1.5406 Å). Data were recorded over a 2*θ* range of 10°–80°, with a step size of 0.02° and a counting time of 2 s per step. The International Centre for Diffraction Data (ICDD) Powder Diffraction File (PDF) cards were used to match the structure of the material with the database. Measurements were performed in reflective mode at 45 kV and 40 mA. Default divergence and anti-scatter slits were employed to optimise analytical conditions.

### Residual stress analysis

Residual stress measurements were performed by XRD using a PANalytical X’Pert^3^ Powder diffractometer, following the sin^2^*ψ* method [23]. Due to the small thickness of the single-layer specimens, destructive techniques such as hole drilling and contour cutting were not suitable for this study. XRD is a widely used non-destructive technique that enables residual stress evaluation without damaging the material, and it is recognized as an effective method for assessing residual stress in LPBF components [24, 25]. Residual stress produces elastic strain within the crystal lattice, which can be determined from changes in the measured lattice spacing. Cu Kα radiation was generated at 45 kV and 40 mA, and a nickel filter was used to suppress Cu Kβ radiation. For Ti6Al4V, the (213) lattice plane, corresponding to a 2*θ* angle of 141.7° is commonly recommended for residual stress analysis because of its suitability for strain measurement with the available X-ray wavelength and instrumental configuration [26]. Accordingly, the diffractometer was programmed to scan over a 2θ range of 138.5510°–143.4486° at each tilt position.

Residual stress was calculated based on the principle that the distortion of the crystal lattice follows a linear elastic relationship, and a diffraction elastic modulus *E*_*213*_ = 110 GPa and Poisson’s ratio *ν*_*213*_ = 0.33 were used for stress evaluation [27]. For each sample, residual stress was measured at the centre of the top surface in both the scanning direction (*σ*_*xx*_) and transverse direction (*σ*_*yy*_). For each direction, diffraction data were collected at seven tilt positions corresponding to sin^2^*ψ* values from 0 to 0.6, equivalent to a *ψ* range of 0°–51°. Peak positions were determined after background, Lorentz–polarisation, and absorption corrections in HighScore Plus, followed by linear fitting of d against sin^2^*ψ* in OriginPro. Each reported stress value was obtained from the slope (*M*) of the linear relationship between lattice spacing (*d*) and sin^2^*ψ* [23]. The X-ray penetration depth was approximately 10 µm, so in this study only the near-surface stress was investigated. No repeated measurements were performed at additional locations on the same specimen, and this limitation was considered when interpreting the quantitative differences between processing conditions.

### Cooling rate simulation

The cooling rates of DAM Ti6Al4V specimens processed with 3, 4, and 5 lasers at scanning speeds ranging from 100 to 250 mm/min were simulated using a transient finite-element thermal model implemented in ANSYS 2021 R2 (ANSYS, Inc., Canonsburg, PA, USA). The model represented the combined thermal input from the simultaneously operating diode lasers and predicted the temperature evolution within the powder-bed domain. Temperature–time data were extracted from a fixed point on the top surface along the centreline of the scanning path, and the cooling rate was determined from the descending portion of the simulated thermal cycle.

A three-dimensional computational domain of 2 × 1 × 0.5 mm^3^ was used. The domain was discretised using uniform cubic elements measuring 15 × 15 × 15 µm^3^. A mesh-independence assessment was performed during model development. The 15 µm mesh was retained because further mesh refinement did not produce an appreciable change in the predicted thermal response but substantially increased the computational cost. The powder bed was represented as a homogeneous continuous medium, and the temperature-independent thermophysical properties of bulk Ti6Al4V (listed in Table 3 [28]) were assigned to the domain. These thermophysical properties were treated as constant values over the simulated temperature range.

**Table 3:** Thermophysical properties of Ti6Al4V used in the finite-element thermal model [28]

Properties	Values (Units)
Solidus temperature	1878 (K)
Liquidus temperature	1923 (K)
Density	4375 (kg m^−3^)
Thermal conductivity	7 (W m^−1^ K^−1^)
Thermal diffusivity	2.9 × 10^–6^ m^2^ s^−1^

The laser input was represented by simultaneously moving Gaussian surface heat sources. Each 808 nm diode laser had a laser power of 5 W, an absorptivity of 0.65 [4], and a measured 1/e^2^ spot diameter of 65 µm. Adjacent beam centres were separated by approximately 76 µm. The resulting combined 1/e^2^ beam widths were 217, 294 and 370 µm for the 3-, 4- and 5-laser configurations, respectively, corresponding to total laser powers of 15, 20, and 25 W.

The substrate was maintained at an ambient room temperature of 293 K, with no additional preheating applied. Thermal radiation was imposed on the top surface of the powder bed, while convective heat loss associated with the shielding gas was applied to the four side walls using a surface-film boundary condition with a heat-transfer coefficient of 10 W m^−2^ K^−1^. Heat was dissipated primarily by conduction through the Ti6Al4V domain towards the substrate and adjacent regions. The latent heat of melting and solidification was not included explicitly through an enthalpy or apparent heat-capacity formulation.

The cooling rate was evaluated from the descending portion of the simulated temperature–time curve. It was defined as the average rate of temperature decrease from the *β*-transus temperature, 1267 K, to the martensitic transformation temperature, 847 K, expressed by Eq. (1):

1 $$Cooling \; rate=\frac{1267-847}{\Delta t}$$

where *Δt* is the time required for the sampling point to cool through this temperature interval.

## Results and discussion

### Top morphology and surface roughness analysis

**Fig. 3:**
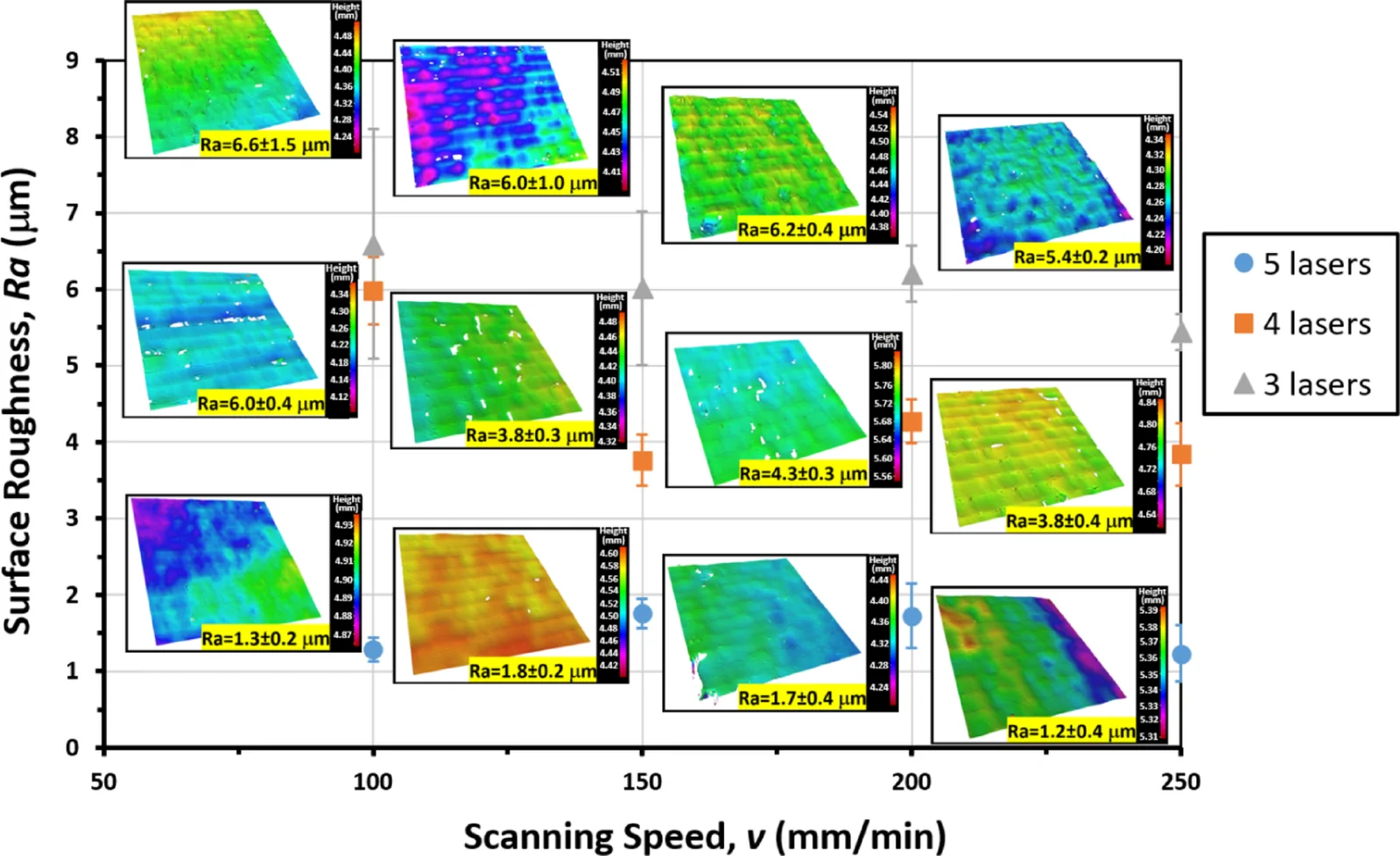
Top-surface morphology and surface roughness (*Ra*) of specimens fabricated with 3 lasers (gray data), 4 lasers (orange data), and 5 lasers (blue data) using varying scanning speeds. 3D surface-height map is shown for each condition, with the height scale bar displayed beside each map

Surface roughness is an important characteristic of additively manufactured components because surface irregularities may act as local stress concentrators. The top surface morphology and roughness of all the DAM specimens are shown in Fig. 3. A three-dimensional surface-height map of the measured central area is presented for each condition. The reported *Ra* value is the average of five measurements, and the error bars represent the corresponding standard deviation.

The measured *Ra* values varied considerably with laser configuration. 5-laser specimens exhibited the smoothest surfaces (*Ra* = 1.2–1.8 µm), followed by the 4-laser condition, while the 3-laser group showed the roughest surfaces (*Ra* = 5.4–6.6 µm). One likely contributor to this trend is the overlap between adjacent melt tracks. As shown in Fig. 2, the combined beam width increased with the number of activated lasers. At a constant hatch spacing, this increased the overlap ratio between successive tracks from approximately 7.8% in the 3-laser condition to 45.9% in the 5-laser condition. The greater overlap may have promoted partial remelting and improved coalescence between neighbouring tracks, thereby reducing local height discontinuities. Marangoni-driven melt flow may also have assisted the redistribution of molten material before solidification. Although this mechanism was not directly examined in the present study, it provides a physically plausible explanation for the more uniform surface morphology observed in the 5-laser condition. The relatively thick 1 mm powder layer may also have influenced the surface morphology by providing limited mechanical support beneath the molten region. Local powder rearrangement and contraction during cooling may therefore have caused partial settlement into the underlying loose powder, contributing to surface waviness and height irregularities.

Figure 3 also indicates a slight reduction in surface roughness with increasing scanning speeds. At constant laser power and hatch spacing, higher scanning speeds reduced the energy input, per length and produced shallower melt pools, which may have limited local surface deformation and height variation. However, the influence of scanning speed was less pronounced than that of laser configuration. The lowest *Ra* values measured in the present study were below the range of 10–15 µm reported for as-built LPBF Ti6Al4V [29], indicating the potential of DAM to produce a relatively smooth surface under the investigated conditions.

### Melt pool observations

**Fig. 4:**
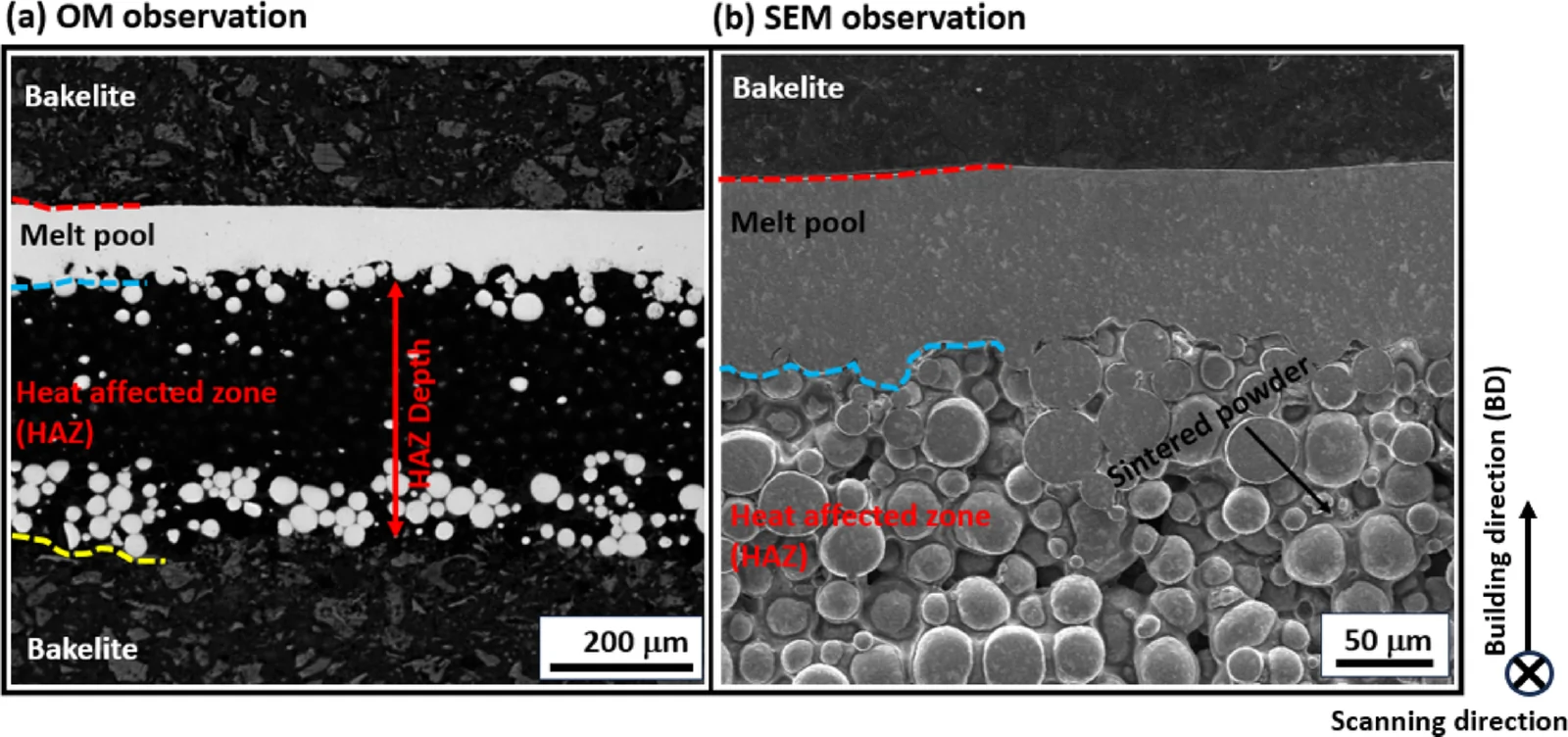
Cross-sectional **a** OM images and **b** SEM images of specimens fabricated with 5 lasers at a scanning speed of 100 mm/min

Representative cross-sectional images of the melt pools are shown in Fig. 4 for the sample processed using 5 lasers at a scanning speed of 100 mm/min. The OM image in Fig. 4a reveals several different regions: the melt pool, the heat affected zone (HAZ), and the surrounding Bakelite mounting material. The higher-magnification SEM image in Fig. 4b shows that the HAZ consists of partially sintered powders bonded together, while the melt pool is fully melted and resolidified. The HAZ appears predominantly dark in the OM image because its porous and irregular structure scatters the incident light, while the numerous interparticle voids provide little reflected signal. At the higher spatial resolution and depth of field provided by SEM, the same region is resolved as partially sintered powder particles joined by local necks and separated by interconnected pores. During the laser irradiation, the powder surface reaches temperatures above the melting point, resulting in complete melting and the formation of melt pools upon solidification. However, heat conducted into the powder bed was insufficient for completed melting. Consequently, loose powders beneath the melt pool undergo partial sintering rather than full consolidation, leading to the formation of the HAZ.

**Fig. 5:**
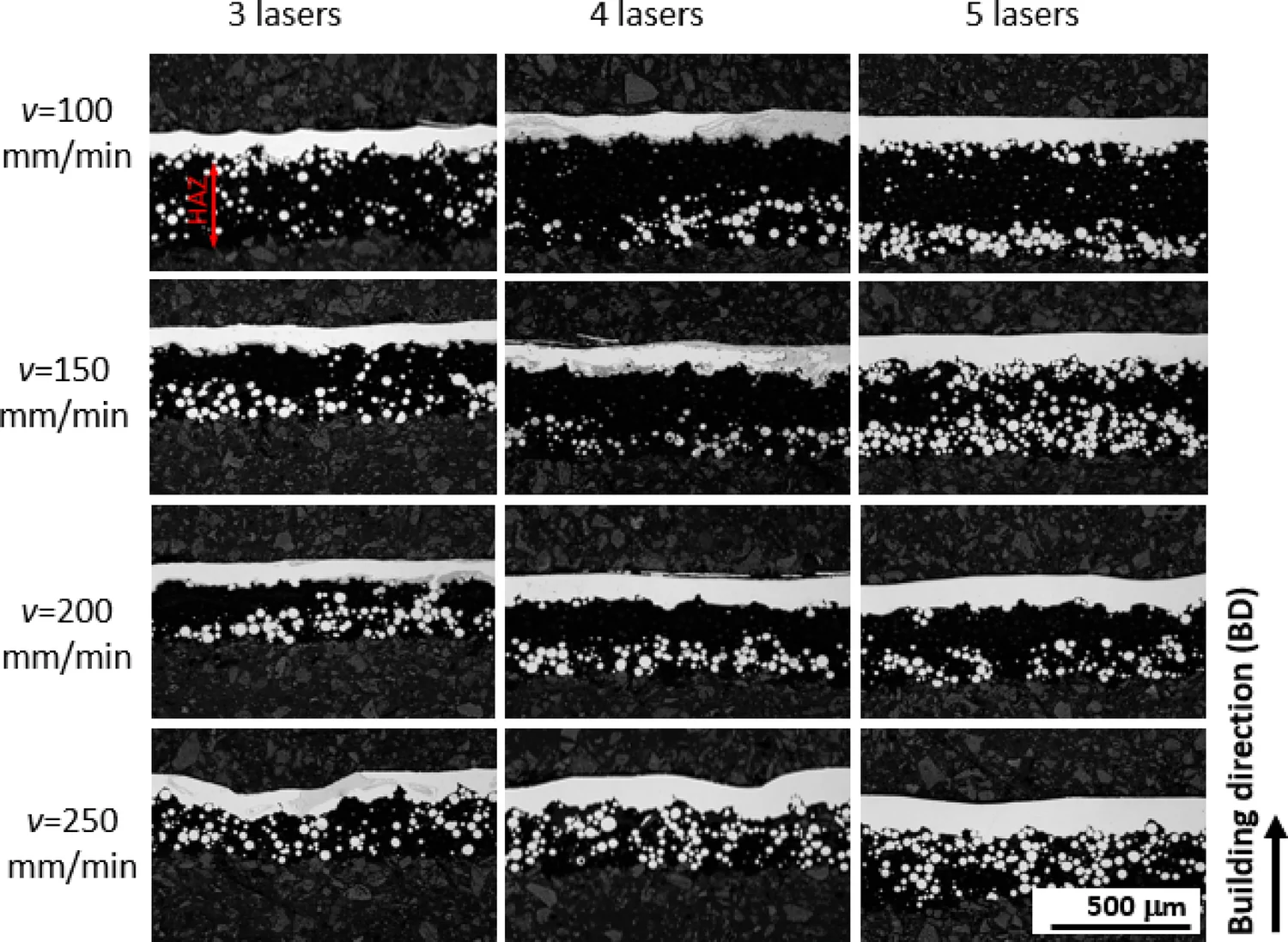
Cross-sectional OM images of specimens fabricated with 3, 4, and 5 lasers using varying scanning speeds

Figure 5 summarises the cross-sectional OM images of all the specimens produced using 3, 4, and 5 lasers at scanning speeds ranging from 100 to 250 mm/min. At the resolution of the OM cross-sections, no obvious large pores, keyhole-shaped defects, or large lack-of-fusion gaps were identified within the melt pools or between adjacent tracks. The representative SEM image in Fig. 4b also shows no clearly visible pores or cracks within the examined melt pool region. Clear differences in melt pool morphology were observed with varying laser configurations and scanning conditions. At a scanning speed of 100 mm/min, the 3-laser samples appeared relatively irregular melt pool shapes compared with the 4- and 5-laser samples. This observation is consistent with higher surface roughness reported in the 3-laser samples, which was associated with the lower overlap ratio between adjacent tracks. Under constant hatch spacing, the limited overlap may have resulted in less uniform remelting and increased local height variations between neighbouring tracks. This feature was most pronounced at lower scanning speeds, where higher nominal areal energy densities promoted more curved molten pools. As the scanning speed increased to 250 mm/min, the melt pools became less stable and more irregular. The higher scanning speed reduced the nominal areal energy density, limiting melt penetration and reducing the degree of melting within the powder bed [30]. The resulting melt pools were shallower and more susceptible to instability associated with insufficient fusion.

**Fig. 6:**
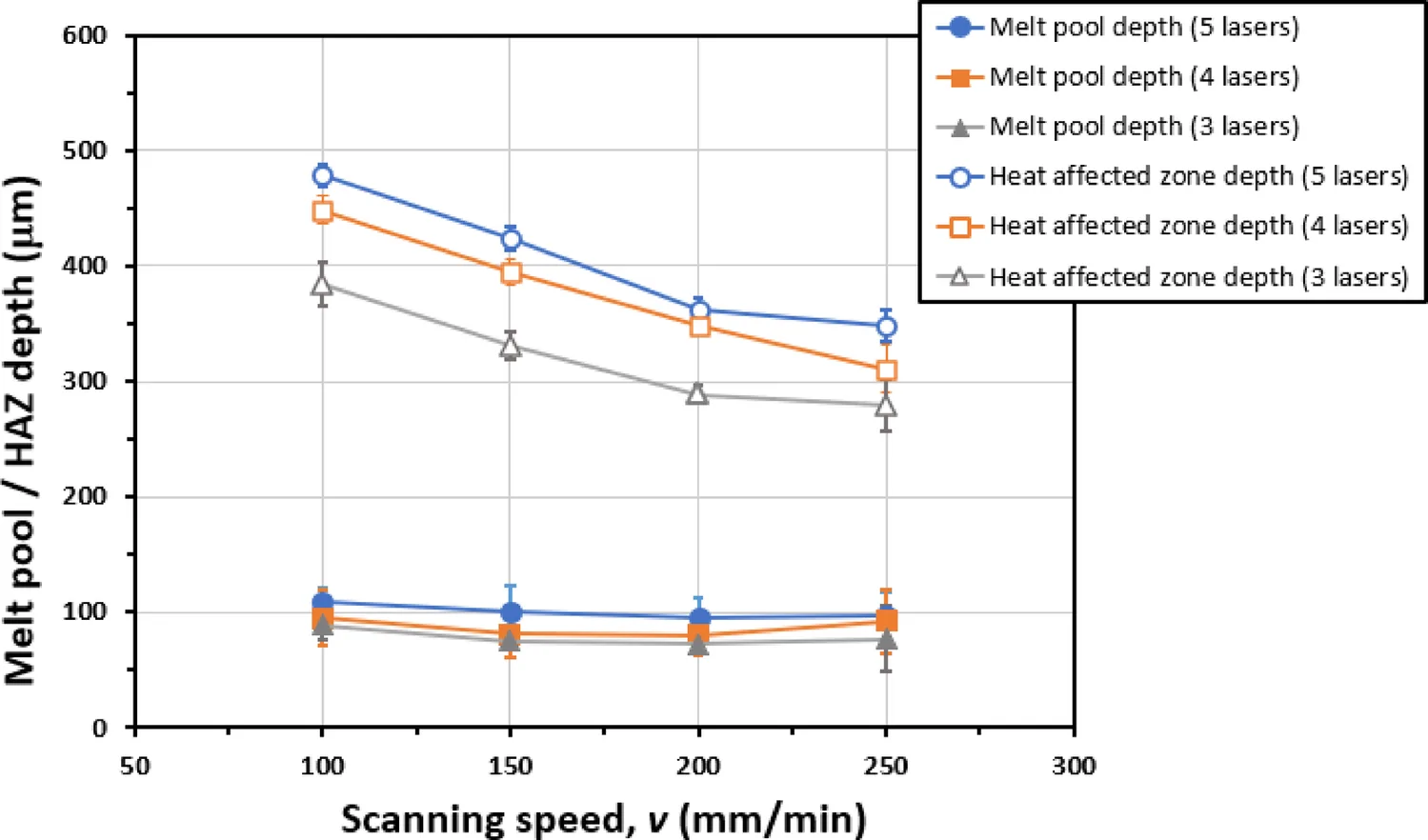
Plot of melt pool depth and heat affected zone vs. scanning speeds for specimens fabricated with 3 lasers (gray data), 4 lasers (orange data), and 5 lasers (blue data)

Figure 6 displays the variation in the melt pool depth and HAZ depth under different laser configurations and scanning conditions. Increasing the number of activated lasers generally increased both melt pool depth and HAZ depth. At a scanning speed of 100 mm/min, the melt pool depth increased by nearly 17% from approximately 92 µm in the 3-laser samples to 108 µm in the 5-laser samples. A similar trend was observed for HAZ depth, which increased from 380 µm to 475 µm. The larger increase in HAZ depth compared with melt pool depth suggests that the broader thermal source and greater track overlap enhanced heat diffusion into the surrounding powder bed more strongly than they increased the depth of the fully molten region.

The total laser power increased from 15 W in the 3-laser configuration to 25 W in 5-laser configuration, while the corresponding combined beam width is increased (217 µm for 3 lasers and 370 µm for 5 lasers). However, the deeper melt pools and HAZ regions observed in the 5-laser samples indicate greater overall thermal input and increased heat accumulation within the material, which is also consistent with the highest nominal areal energy densities obtained in 5-lasers condition. For a given number of activated lasers, a lower scanning speed of 100 mm/min produced deeper melt pools and HAZ. In the 5-laser configuration, increasing scanning speed from 100 to 250 mm/min reduced the HAZ depth from approximately 475 µm to 345 µm, and the melt pool depth from around 108 µm to 95 µm. This indicates that scanning speed had a stronger influence on heat diffusion into the surrounding powder bed than on the fully molten region itself. The reduced thermal penetration at higher scanning speeds is attributed to the shorter laser–material interaction time and lower nominal areal energy densities.

### Phase analysis

**Fig. 7:**
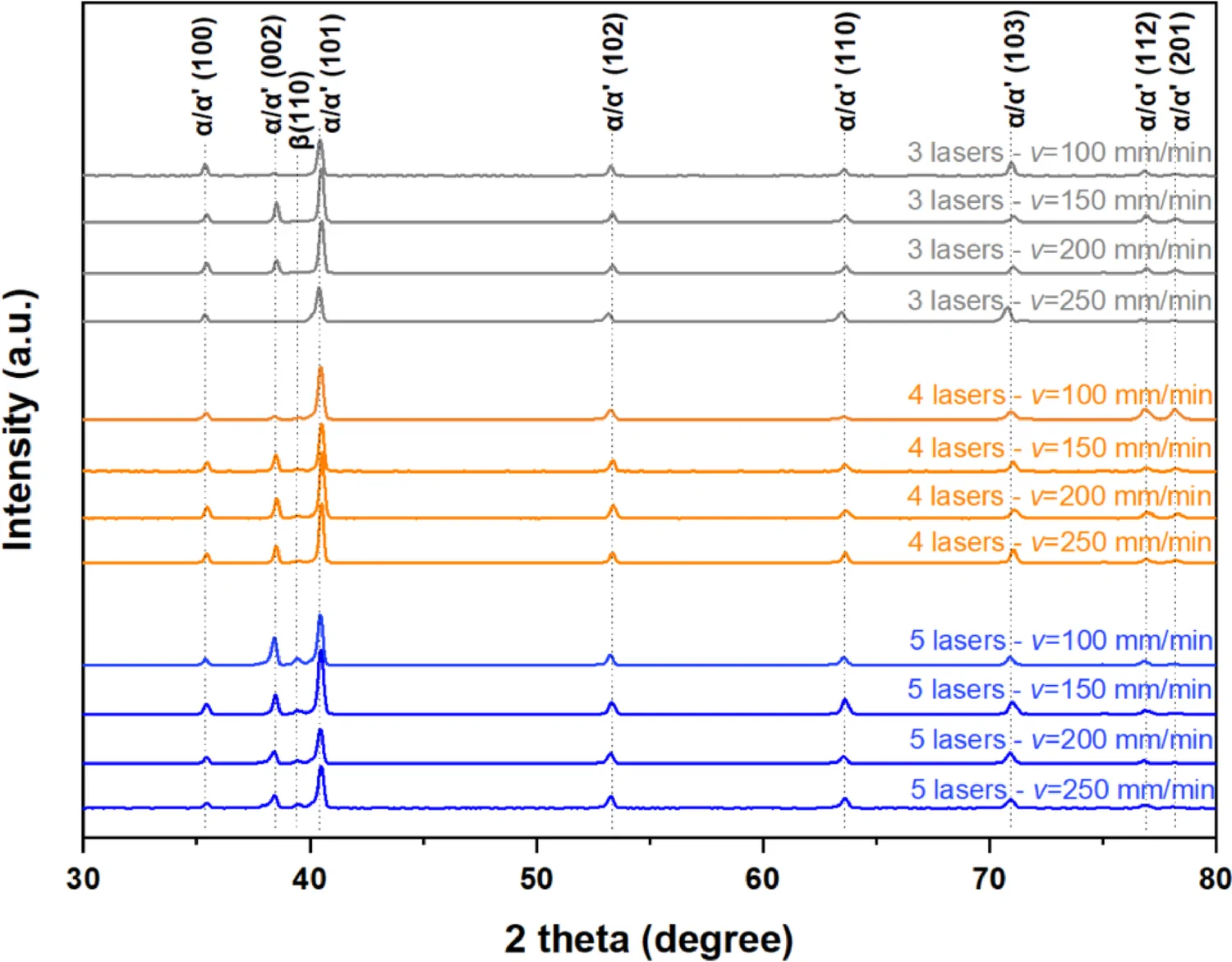
XRD phase identification for specimens fabricated with 3 lasers (gray data), 4 lasers (orange data), and 5 lasers (blue data) using varying scanning speeds

Ti6Al4V is a dual-phase titanium alloy consisting primarily of a hexagonal close-packed (HCP) *α* phase, and a body-centred cubic (BCC)* β* phase [31]. XRD was used to identify the phase constitution of DAM Ti6Al4V specimens fabricated with 3, 4, and 5 lasers at scanning speeds ranging from 100 to 250 mm/min. The corresponding diffraction patterns are presented in Fig. 7. The dominant reflections were indexed to the HCP *α*/*α’* structure. However, the *α* and *α*′ phases could not be reliably distinguished by conventional XRD due to their closely related crystal structures and similar lattice parameters.

In addition to the dominant *α*/*α’* matrix, a weak diffraction peak assignable to retained *β*-Ti was detected at approximately 2*θ* = 39.5° in 4- and 5-laser specimens. The presence of retained *β* is consistent with the lower simulated cooling rates associated with these processing conditions. In LPBF Ti6Al4V, very high cooling rates generally suppress diffusion-controlled phase transformations, promoting the formation of an *α*′-type martensitic microstructure and limiting the retention of *β* phase [32]. In contrast, the lower cooling rates in DAM may provide more time for elemental partitioning and transformation during cooling, thereby increasing the tendency for *β* phase to be retained at room temperature [33].

The *β*-related reflection was observed in the 4- and 5-laser specimens but was not clearly detected in the 3-laser condition. It was most pronounced at the lowest scanning speed of 100 mm/min and gradually weakened with increasing scanning speed. This trend is consistent with the larger melt pools, deeper thermal penetration, and lower simulated cooling rates associated with the higher-energy processing conditions. At a given scanning speed, increasing the number of activated lasers increased the nominal areal energy, whereas higher scanning speeds shortened the interaction time and were associated with weaker *β*-related diffraction peaks. Owing to the weak and partially overlapping nature of the *β*-related reflection, reliable quantitative phase analysis could not be performed. The observed variation is therefore interpreted only as a qualitative trend in the *β*-related diffraction response and not as a quantitative measurement of the retained *β* phase fraction.

### Grain structure

**Fig. 8:**
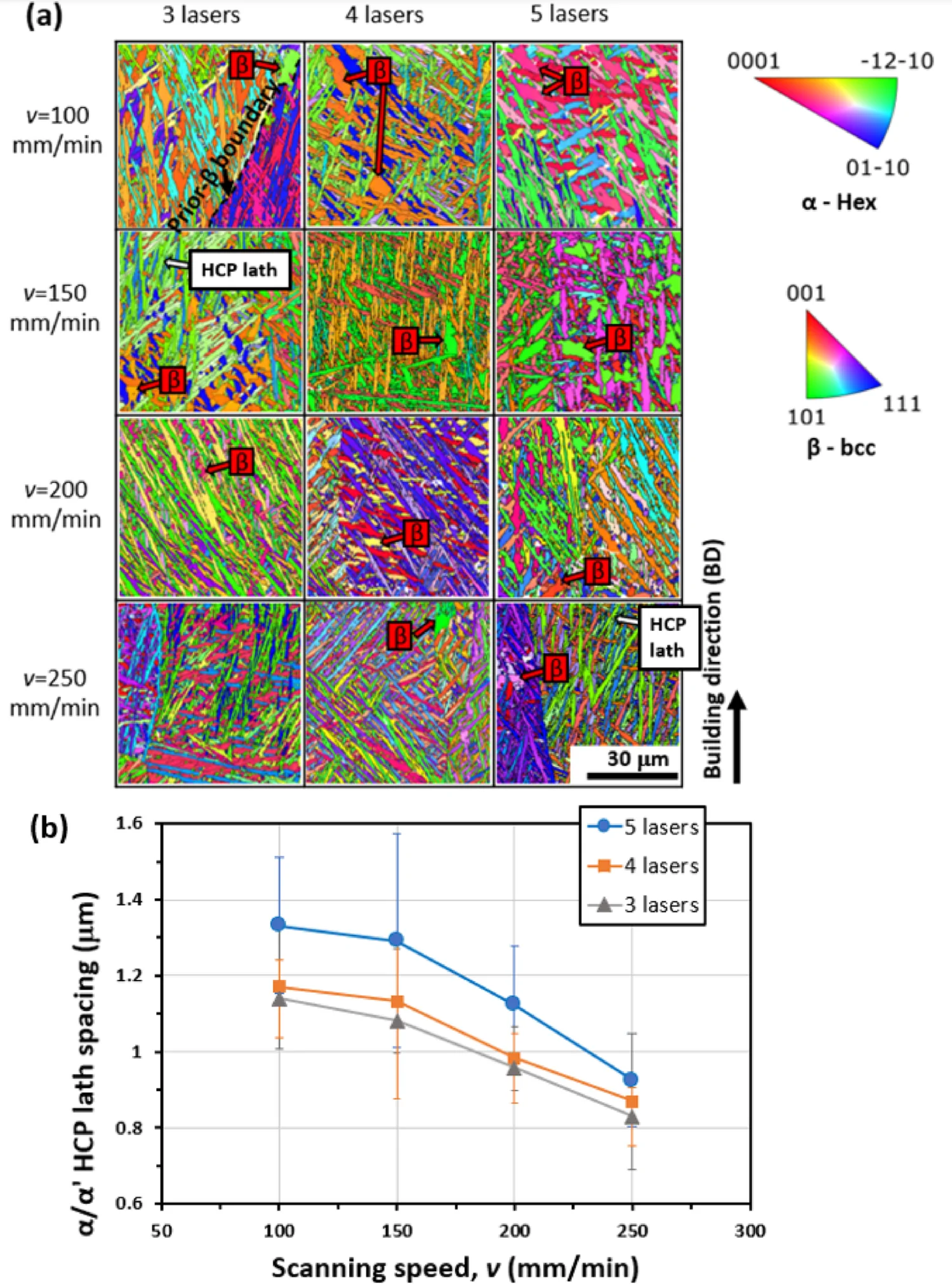
**a** EBSD orientation maps with respect to building direction (with a step size of 0.1 µm at 650 × magnification); **b** HCP lath spacing for specimens fabricated with 3 lasers (gray data), 4 lasers (orange data), and 5 lasers (blue data) using varying scanning speeds

EBSD inverse pole figure (IPF) maps of all specimens are shown in Fig. 8a, where different colours represent different crystallographic orientations. The microstructure was dominated by the Widmanstätten basket-weave morphology consisting of fine acicular HCP laths transformed from the parent *β* phase. Clusters of similarly oriented HCP laths were considered to have originated from individual prior-*β* grains [34]. Coarse columnar prior-*β* grain boundaries were visible in several maps, an example is marked by a black dashed line in the 3-laser specimen processed at 100 mm/min (top left corner). The orientation contrast across these boundaries reflects the different *α*/*α*′ variants inherited from neighbouring prior-*β* grains during cooling.

A fine acicular HCP lath structure was observed in all EBSD maps. However, the individual HCP laths could not be assigned specifically to *α* or *α*′ because these phases have closely related crystallographic structures. The regions indexed as retained *β* were located mainly along prior-*β* grain boundaries or between HCP laths, as indicated by the red arrows. Such regions were observed more frequently in the 4- and 5-laser specimens, particularly at lower scanning speeds. This observation is consistent with the *β*-related peaks detected by XRD in specimens processed under the higher nominal areal energy densities and lower simulate cooling rates. However, the EBSD step size of 0.1 µm was not sufficient to resolve nanoscale film-like retained *β* between individual laths. Consequently, the phase maps show only *β* regions large enough to be spatially resolved and successfully indexed, and the absence of indexed *β* does not confirm its complete absence.

Few regions indexed as retained *β* were observed in the 3-laser specimen processed at 250 mm/min. The higher cooling rate and shorter thermal exposure under this condition may have limited elemental partitioning and reduced the amount of β retained after cooling, resulting in a finer acicular HCP microstructure. Similar observations of retained *β* in DAM-processed Ti6Al4V have been reported previously [21]. Previous studies have shown that *α*-lath structure significantly affects mechanical behaviour of Ti6Al4V. Finer laths increase dislocation boundary density, which may prevent the dislocation motion and contribute to higher strength and hardness [35, 36].

In this study, the measured acicular HCP lath spacings are displayed in Fig. 8b. For all laser configurations, the lath spacing decreased with increasing scanning speeds. In 5-laser specimens, the average lath spacing decreased from approximately 1.33 µm at 100 mm/min to 0.93 µm at 250 mm/min, corresponding to a reduction of about 30%. Similar reductions of approximately 26% and 27% were observed for the 4- and 3-laser configurations, respectively. The refinement of HCP laths at higher scanning speeds is consistent with the corresponding increase in simulated cooling rate. Faster cooling increases undercooling and limits the time available for diffusional transformation and lath growth, resulting in a finer acicular *α*/*α*′-type HCP lath microstructure. A systematic effect of laser configuration was also observed. At a given scanning speed, the 5-laser specimens exhibited the coarsest laths, whereas the 3-laser specimens showed the finest microstructure. This trend closely follows the variation in melt pool and HAZ dimensions shown in Fig. 6. Samples exhibiting greater thermal penetration generally produced coarser laths, indicating a longer time available for microstructural development during cooling.

Although the scanning area increased with the number of activated lasers, the higher total laser power and greater overlap ratio in 5-laser configuration resulted in localized heat accumulation and lower cooling rate, thereby promoting lath coarsening. Similar relationships between thermal conditions and *α*-lath spacing have been reported in other additive manufacturing processes. Scharowsky et al. [37] demonstrated that variations in energy input during electron beam melting (EBM) affect lamellar spacing due to variations in the solidification parameters, particularly the temperature gradient (*G*) and solidification front rate (*R*). Chen et al. [38] reported *α*-lath spacing of 0.7 ± 0.24 µm near the top surface of LPBF Ti6Al4V specimens. Another study reported average *α*-lath widths of 0.3 µm in as-built LPBF Ti6Al4V [39]. The larger lath spacings measured in the present study are consistent with the substantially lower cooling rates associated with the DAM process.

### Cooling rate simulation

**Fig. 9:**
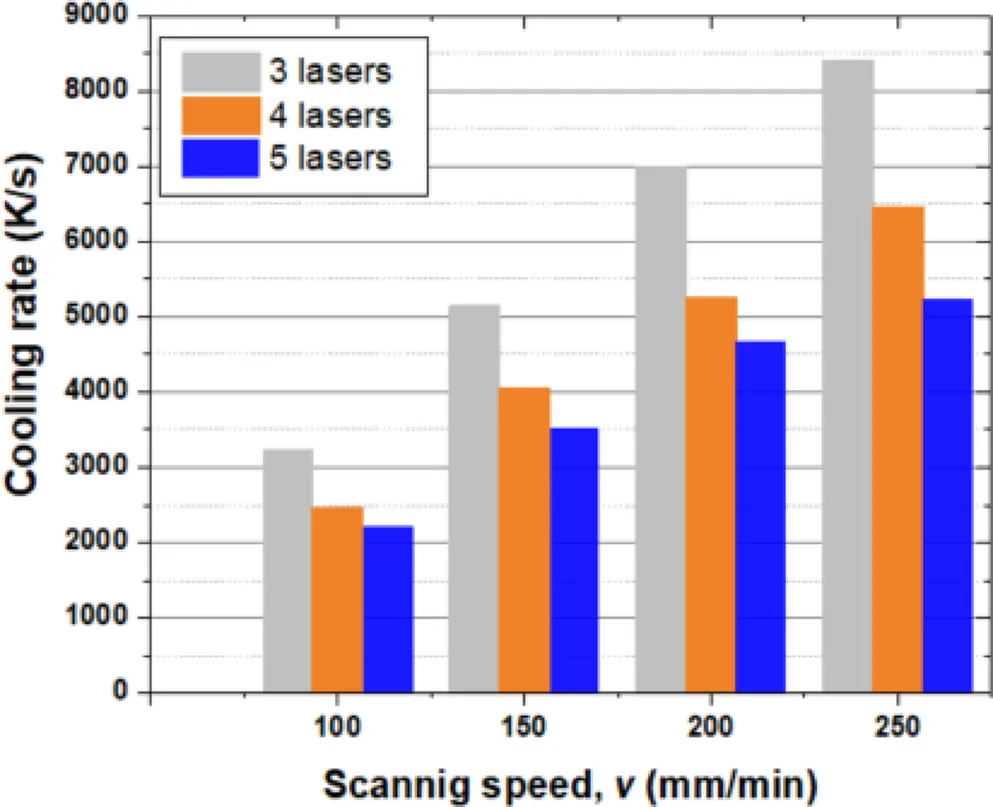
Predicted cooling rates for specimens fabricated with 3 lasers (gray data), 4 lasers (orange data), and 5 lasers (blue data) using varying scanning speeds

The comparatively coarser HCP laths observed in DAM specimens are attributed to lower cooling rates relative to LPBF, which may allow greater time for diffusional growth and lath coarsening. The simulated cooling rates for all processing conditions are presented in Fig. 9. The highest cooling rate of 8400 K/s was obtained at a scanning speed of 250 mm/min with 3 lasers, whereas the lowest cooling rate of 2211 K/s was recorded at 100 mm/min with 5 lasers. Although the cooling rates increased substantially with scanning speed and decreased with the number of activated lasers, all DAM conditions produced cooling rates considerably lower than those typically reported for LPBF Ti6Al4V, which can exceed 4 × 10⁷ K/s [40].

For a given laser configuration, increasing the scanning speed from 100 to 250 mm/min approximately doubled the simulated cooling rate. This trend is attributed to the shorter laser-material interaction time and lower nominal areal energy density associated with faster scanning. In contrast, increasing the number of activated lasers produced a decreasing trend in the simulated cooling rate. The 5-laser configuration generated larger melt pools and deeper thermal penetration, as shown in Fig. 6. The combined effects of higher total nominal power, a broader multi-beam heat source, and greater geometric track overlap were associated with a longer thermal cycle and more gradual cooling through the post-solidification transformation range.

The cooling rate trends are consistent with the microstructural observations in Fig. 8. Conditions associated with lower cooling rates generally produced coarser HCP laths and more pronounced *β*-phase retention, whereas higher cooling rates promoted finer HCP lath structures. For example, the 5-laser specimen processed at 100 mm/min exhibited the lowest cooling rate of 2211 K/s and the largest lath spacing of 1.33 µm, while the 3-laser specimen processed at 250 mm/min showed the highest cooling rate of 8400 K/s and the finest lath spacing of 0.82 µm. These observations support a relationship between the predicted thermal history and microstructural evolution in DAM-processed Ti6Al4V. However, as the FEM model was not directly validated against melt-pool or HAZ dimensions under identical multi-laser conditions, the calculated cooling rates should be regarded as comparative indicators rather than experimentally validated absolute values.

### Residual stress

**Fig. 10:**
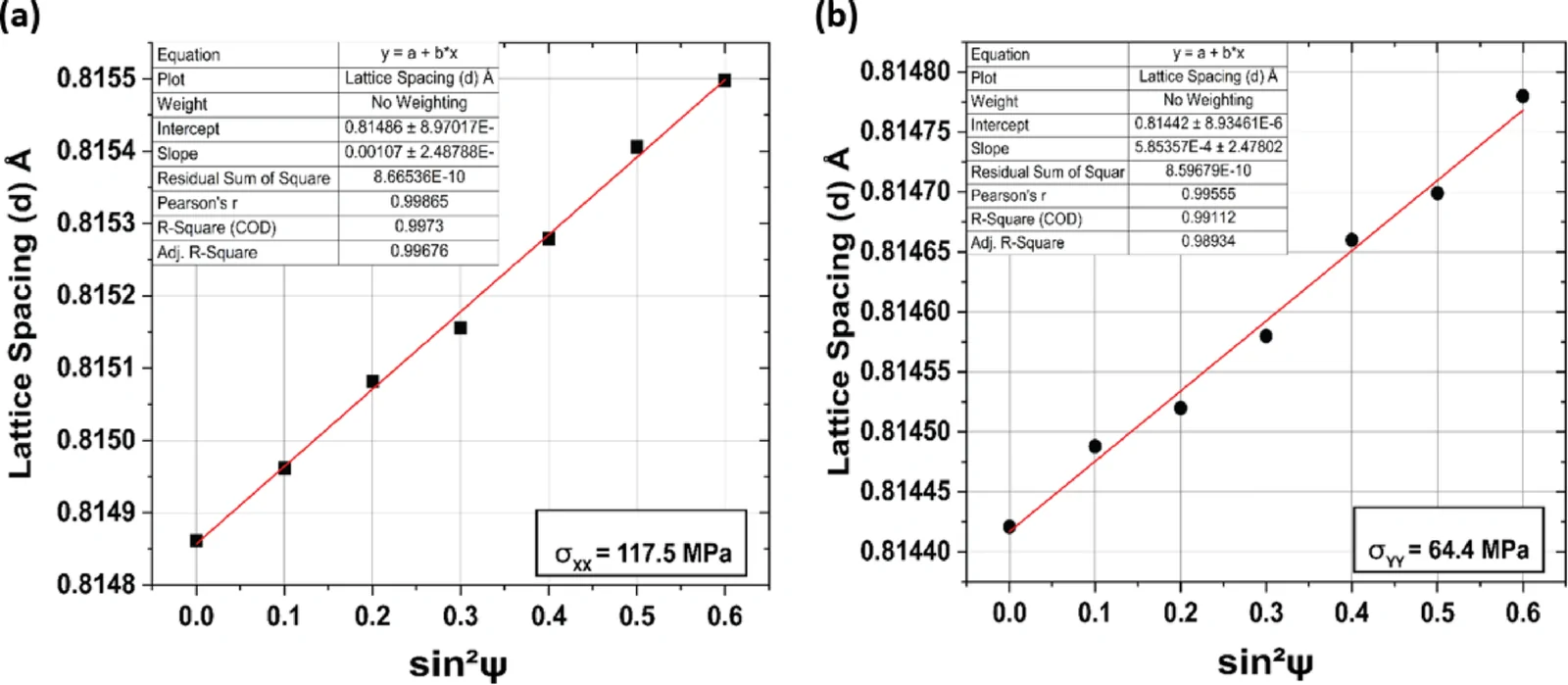
Representative linear fits of lattice spacing, *d*, against sin^2^*ψ* for the specimen processed using 5 lasers at a scan speed of 250 mm/min: **a** scanning direction, and **b** transverse direction [41]

Residual stress was determined from the linear relationship between lattice spacing between lattice spacing, *d*, and sin^2^*ψ*. For each measurement direction, diffraction data were collected at seven *ψ* tilt positions corresponding to sin^*2*^*ψ* values from 0 to 0.6. Representative *d*-sin^*2*^*ψ* fits for the specimen processed using 5 diode lasers at a scan speed of 250 mm/min are shown in Fig. 10. Figure 10a presents the fit in the scanning direction, with a *R*^*2*^ value of 0.9973, while Fig. 10b presents the fit in the transverse direction, with a *R*^*2*^ value of 0.9911. These plots indicate good linearity for the selected representative measurements.

**Fig. 11:**
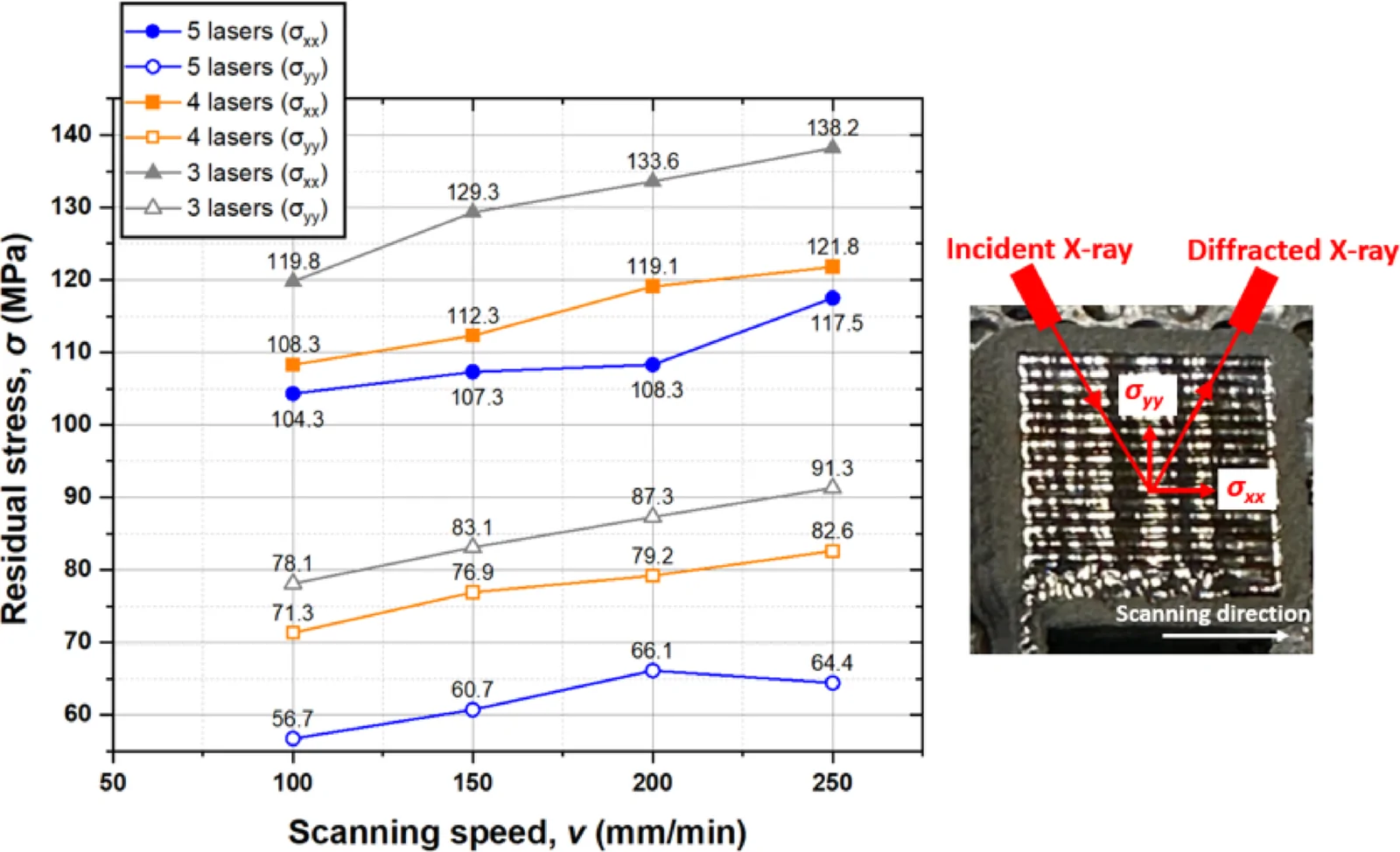
Residual stress in *σ*_*xx*_ direction (parallel to the scanning direction) and *σ*_*yy*_ direction (perpendicular to the scanning direction) for specimens fabricated with 3 lasers (gray data), 4 lasers (orange data), and 5 lasers (blue data) using varying scanning speeds

Residual stresses measured by XRD are presented in Fig. 11 (left), and the schematics of measurements directions *σ*_*xx*_ and *σ*_*yy*_ are illustrated in Fig. 11 (right). For all processing conditions, residual stresses measured along the scanning direction (*σ*_*xx*_) are consistently higher than those measured perpendicular to the scanning direction (*σ*_*yy*_), with an average increase of approximately 53%. Similar anisotropic residual stress distributions have been reported for LPBF-fabricated Ti6Al4V [42, 43]. The higher stresses along the scanning direction may be associated with the directional thermal history during the laser scanning. Continuous energy deposition promotes the elongated melt pools, while thermal contraction during cooling is constrained by neighbouring solidified material, generating tensile stresses along the scan path. In contrast, regions perpendicular to the scanning direction experience repeated thermal exposure from adjacent tracks, which may contribute to partial stress relaxation and reduce the magnitude of *σ*_*yy*_.

A clear influence of laser configuration was observed in the measured near-surface residual stress. The 3-laser specimens showed the highest values (up to 138.2 MPa in the *σ*_*xx*_ direction and 91.3 MPa in the *σ*_*yy*_ direction). Residual stress exhibited a decreasing trend with increasing laser number, with the lowest values observed in the 5-laser specimens (up to 117.5 MPa in the *σ*_*xx*_ direction and 66.1 MPa in the *σ*_*yy*_ direction). Relative to the 3-laser condition, the maximum *σ*_*yy*_ decreased by approximately 28%, whereas the maximum *σ*_*xx*_ decreased by about 15%. This decreasing trend with increasing laser number is broadly consistent with the larger melt pools, deeper thermal penetration, and lower simulated cooling rates observed in the 4- and 5-laser configurations.

Scanning speed also influenced the measured residual stress response. For all laser configurations, residual stress generally increased with scanning speed, with an overall increase of approximately 12.5–16.9% as the scanning speeds rose from 100 to 250 mm/min. The higher simulated cooling rates at faster scanning speeds (Fig. 9) are expected to increase the thermal contraction mismatch between the molten region and the surrounding solidified material, promoting higher tensile residual stresses. A similar association between cooling rate and tensile residual stress has been reported for LPBF-processed Ti6Al4V [44].

These trends of residual stress should be considered alongside the differences in surface conditions and microstructure between specimens. The 3-laser samples had the roughest surfaces, with *Ra* values of approximately 5.4–6.6 µm, whereas the 5-laser samples were considerably smoother, with *Ra* values of approximately 1.2–1.8 µm. At higher *ψ* angles, surface asperities may alter the effective diffraction geometry and introduce peak broadening or asymmetry, which can affect the fitted peak position and hence the slope of the *d*-sin^2^*ψ* relationship. The greater roughness of the 3-laser specimens may therefore have contributed to some of the higher measured stress and scatter. However, the direction of this bias is dependent of the local measured surface geometry.

The EBSD results also showed differences in crystallographic orientation and HCP lath spacing across the processing conditions. Texture and grain orientation affect the population of grains contributing to the selected *α*-Ti (213) reflection and may affect diffraction intensity and the linearity of the *d*-sin^2^*ψ* fit. The finer lath structure observed under conditions associated with higher simulated cooling rates is consistent with the rapid solidification and transformation behaviour commonly reported for additively manufactured Ti6Al4V. In contrast, the coarser HCP laths measured in the 5-laser specimens indicate a less severe cooling history and greater cumulative thermal exposure. Such thermal conditions may provide more opportunity for strain accommodation and stress relaxation, leading to a lower measured residual stress [45]. However, the effects of lath spacing, thermal history, and surface condition cannot be separated quantitatively in the present study.

The retained *β* phase observed in the 4- and 5-laser specimens may also have influenced the measured stress response. Its presence reflects the greater areal energy density and slower cooling in these conditions, which may have allowed more stress relaxation during cooling. The *α* and *β* phases can also carry strain differently because of their different elastic properties. As the stress calculation was based on the *α*-Ti (213) reflection, the reported values represent the near-surface stress in the *α/α’* phase rather than the average stress of the full* α*+*β* microstructure. Part of the lower measured stress in the 4- and 5-laser specimens may therefore be related to phase-specific strain partitioning as well as their lower cooling rates.

Although residual stress was measured once by XRD based on the sin^2^*ψ* method, the measured values showed a systematic trend with laser number and scanning speed and were correlated well with the simulated cooling behaviour and the observed microstructural evolution. The agreement among these independent indicators provides converging evidence supporting the reliability of the observed stress trends, despite the limited statistical sampling.

It should also be noted that the present work employed a single-layer configuration deposited onto a 1 mm powder bed. Under these conditions, residual stress development is dominated by intra-layer thermal gradients and shrinkage constraints between neighbouring melt pools. The influence of substrate restraint and inter-layer stress accumulation, which are major contributors to residual stress in multi-layer LPBF builds, is therefore minimised. Consequently, the stress values reported here primarily reflect the intrinsic thermal response of the DAM process rather than stress accumulation associated with bulk component fabrication.

## Conclusion

This study investigated the processing of Ti6Al4V alloys using an 808 nm multiple diode laser system and examined the influence of laser number and scanning speeds on surface roughness, melt pool dimensions, grain structure, simulated cooling rate, and residual stress. The key summaries follow below:

- Continuous melt tracks were produced under the investigated single-layer processing conditions using arrays of three, four and five 808 nm diode lasers.
- The measured surface roughness ranged from 1.2 to 6.6 µm, with the 5-laser conditions producing the lowest values. The smoother surfaces were associated with the greater overlap between neighbouring scan tracks, which may have promoted partial remelting and improved track coalescence.
- Increasing the number of activated lasers and reducing the scanning speed were generally associated with lower simulated cooling rates, coarser HCP laths and a greater tendency for retained *β*.
- Near-surface tensile residual stress in the single-layer DAM Ti6Al4V specimens reached a maximum of 138.2 MPa and generally exhibited a decreasing trend with increasing laser number and decreasing simulated cooling rate.
- The stress accumulation, densification, and mechanical properties in multi-layer components require further investigation.

Overall, increasing the number of activated diode lasers was associated with lower simulated cooling rates, lower measured surface roughness and a decreasing trend in near-surface tensile residual stress under the investigated single-layer conditions. However, the present study was limited to single-layer DAM specimens. Further multilayer experiments are required to evaluate cumulative residual-stress development, distortion, process stability, internal densification and mechanical properties under more representative fabrication conditions.

## Data Availability

The data that support the findings of this study are available from the corresponding author upon reasonable request.
